# Revisiting the link between hypertension and hemifacial spasm

**DOI:** 10.1038/srep21082

**Published:** 2016-02-19

**Authors:** Jia-Li Leong, Hui-hua Li, Ling-Ling Chan, Eng-King Tan

**Affiliations:** 1 National University of Singapore, Yong Loo Lin School of Medicine, Singapore, 119077, Singapore; 2 Singapore General Hospital, Health Services Research and Biostatistics Unit, Division of Research, Singapore, 169608, Singapore; 3 Singapore General Hospital, Departments of Diagnostic Radiology and Neurology, National Neuroscience Institute, Singapore, 169608, Singapore; 4 Duke NUS Graduate Medical School, Singapore, 169857, Singapore

## Abstract

The relationship between hypertension and hemifacial spasm (HFS) has been debated. Microvascular decompression surgery is effective in some HFS patients with uncontrolled hypertension. To address current gaps in knowledge, we conducted a meta-analysis of case-control studies that have examined the prevalence of hypertension in HFS patients compared to non-HFS controls. We also evaluated the implications and limitations of the pooled studies. We identified 62 studies from PubMed, The Cochrane Library, Web of Science and Scholar.google.com and six studies that fit our inclusion criteria were included. A random-effects model was used to derive the pooled estimate of the Odds Ratio. The data was plotted on a Forest plot. A pooled analysis involving 51585 subjects, 549 cases, 720 neurological controls and 50316 controls from the general population, showed that HFS patients had a higher chance of developing hypertension (OR = 1.72, 95% CI = (1.12, 2.31), p-value  <0.001). The prevalence of hypertension was higher in HFS patients as compared to non-HFS patients. This meta-analysis highlights a positive correlation between hypertension and HFS. Blood pressure should be closely monitored during the follow-up of HFS patients. Preliminary links between ventrolateral medullary (VLM) compression and HFS should be further evaluated in future studies.

Hemi-facial spasm (HFS) is characterized by intermittent, involuntary contraction of facial muscles, usually unilaterally, though it can affect both sides^1^. It is postulated to be due to a compression, irritation or injury of the facial nerve, anywhere along its course after its exit from the pons^2^ (Fig. 1).

Hypertension is a relatively common ailment among the general population. Based on results from the National Health Survey in Singapore^3^, almost 1 in 4, between the ages of 18 to 69 suffer from hypertension. This prevalence increases significantly to 53.4%, i.e. more than 1 in 2, between the ages of 60 to 69.

Hypertension has long been suspected as having a cause and effect relationship with HFS, and studies have been conducted to look into this relationship but their findings have been debated. A meta-analysis of properly conducted case control studies will help to address this relationship. Hence, we did a meta-analysis of the case-control studies that have investigated the relationship between hypertension and HFS and evaluated the challenges and limitations of these studies.

## Results

### Demographics of participants

A total of 549 HFS cases and 720 neurological controls contributed to the meta-analysis. Sandell *et al.*^4^ used the general population as the control group via data from 50316 participants in the Nord-Trondelag Health Study 3 (HUNT3). The background information of the participants is presented in Table 1.

The mean sample size per study for cases was 91.5 while that for neurological controls was 144. The overall gender percentages for males were 33.2% and 32.1% for cases and controls respectively. In cases, the overall age range was from 20 to 83 years old, while in controls, the age range was from 20 to over 90 years old. Within each study, apart from the study by Sandell *et al.*, all controls were age- and gender-matched and there were no significant statistical differences in demographics. The study by Sandell *et al.* did, however, account for age and gender when comparing the data. Participants with secondary causes of hemifacial spasm and hypertension were also excluded from the study to reduce confounding effect. Notably, the studies by Tan *et al.*^5^ and Nakamura *et al.*^6^ were based on Asian populations in contrast to Caucasian populations in the other studies.

### Pooled Analysis

**Figure 2:**
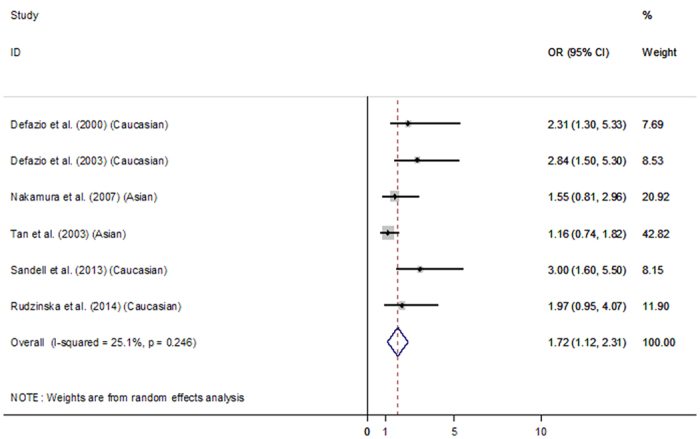
Analysis of pooled published studies.

Given the moderate heterogeneity found by *I*^*2*^-statistic, pooled estimate of Odds Ratio was derived using a random effects model to account for heterogeneity. The pooled result by means of a random effects model showed that patients had a high chance of developing hypertension (OR = 1.72, 95% CI = (1.12, 2.31), p-value  <0.001). This is presented on the Forest Plot (Fig. 2) below. The results showed that the prevalence of hypertension was higher in HFS patients as compared to controls.

### Description of Individual Results and Limitations

All the individual studies produced an Odds Ratio of more than 1.0, for the prevalence of hypertension in cases versus controls. In the Asian studies (Nakamura *et al.* and Tan *et al.*) this was not found to be statistically significant, since the 95% confidence interval straddles 1. However, as the sample size is small, we did not sub-analyse based on ethnicity.

In addition, all studies^4^,^5^,^6^,^7^,^8^,^9^ found a correlation between the prevalence of arterial hypertension in cases and the presence of neurovascular compression. 3 out of 6 found that this was more so in patients with left-sided symptoms while the other 3 studies did not specifically differentiate left- and right-sided disease.

The 6 studies^4^,^5^,^6^,^7^,^8^,^9^ we have chosen for our meta-analysis have inherent differences that must be considered. These include:

1. Study subjects of different ethnicities
2. Definition of hypertension
3. Inclusion Criteria of the study

The studies’ subjects were of different ethnicities. Four of the papers studied Caucasian subjects while 2 of them studied Asian subjects. Due to the small number of papers, we did not sub-analyse the data based on ethnicity, but we expect that there may be a significant difference between results from each ethnic group.

Four studies defined hypertension as an average systolic BP ≥ 140 mmHg and/or diastolic BP ≥ 90 mmHg. The average was taken from at least 2 readings per visit taken at least 2 minutes apart, and these readings were taken on at least 2 separate visits over maximum one month. 1 study defined it as systolic BP ≥ 160 mmHg and/or diastolic BP ≥ 95 mmHg based on readings from 3 separate visits over 2 to 3 weeks. 1 study classified patients as hypertensive if they had been previously diagnosed – diagnostic criteria not specified – and started on anti-hypertensive medications. These differences in definitions could result in an over- or underestimation of the prevalence of hypertension in some of the papers.

While the inclusion criteria for the HFS cases were similar in all the studies, 5 studies recruited patients without HFS from the same referral centre, and matched for age and gender, as controls, while 1 study used the data of the general population obtained from the Nord-Trondelag Health Study 3 (HUNT3).

The heterogeneity of the study populations may also suggest that there is no “one size fit all” intervention we can apply, and each population would still need to streamline the interventions to best fit their target population^9^. Care should be taken before extrapolating the results to a specific population.

## Discussion

The results of the meta-analysis showed that there is indeed a higher prevalence of hypertension among HFS patients as compared to non-HFS controls. These findings bring about clinical implications.

In patients presenting with HFS without hypertension, we need to have a high index of suspicion that hypertension may be present at diagnosis or develop in the course of the illness. Therefore, regular blood pressure monitoring should be performed during follow-up visits for early detection and intervention of hypertension should it develop^10^.

In addition, in patients with hypertension but no HFS, we also need to consider that HFS may develop. Although hypertension is not a clear risk factor for HFS, a study by Rudzinska *et al.* found that an increased duration of arterial hypertension was a significant risk factor for HFS, suggesting a possible etiologic link^11^. Perren F *et al.*^12^ found a relationship between hemodynamic changes in the vessels most frequently associated with HFS and the side of the HFS and arterial hypertension, was found in 58% of their patients. Oliveira *et al.*^13^ found hypertension to be frequent in patients with HFS (67%) in an uncontrolled study.

Since HFS is a very uncommon disease compared to hypertension, the risk of developing HFS in a hypertensive patient is still low, it may seem overly cautious to warn every patient with hypertension about HFS. However, it may be good to educate the general practitioner on this link so they can keep a watch out for the patients under their care. Future studies should address if controlling hypertension can help in managing HFS.

The link between HFS and hypertension brings ventrolateral medullary (VLM) compression into question. A meta-analysis done by Boogaarts *et al.* demonstrated a positive correlation between hypertension and compression of the left-sided medulla oblongata, suggesting a possible etiologic link between VLM compression and hypertension^14^. However, there is limited literature on the relationship between VLM compression and HFS. 2 studies studied the volume of the posterior cranial fossa (PCF) space since a smaller volume predisposes to neurovascular compression^13^,^15^ and found that the size of the PCF space was smaller in HFS patients compared to controls, regardless of whether the HFS patients had hypertension or not, though in 1 study the results were statistically insignificant. Microvascular decompression (MVD) surgery has proven to be an effective intervention in the management of some cases HFS^16^. Whether MVD should be considered in HFS patients with uncontrolled hypertension and VLM compression needs to be further evaluated.

Current case-control studies in HFS and hypertension have limitations. Their cross-sectional design only allowed the prevalence to be measured from a single point in time. A prospective cohort study with less bias and confounding factors may provide additional clues on the cause and effect association^17^. There is also a bias against negative association studies and certainly publication of such studies is encouraged. Ideally, the comparison should be made between HFS due to neurovascular conflict and subjects having neurovascular conflict without HFS. However, imaging data are not available for all the included studies for us to make any meaningful analysis. HFS patients without HTN should be followed-up to see if hypertension develops later on, and if the severity of HFS increases as a result. In those with pre-existing hypertension, further studies could demonstrate if the severity of HFS fluctuates with fluctuations in BP. For the controls, the etiologic link between hypertension and HFS could be further studied by assessing if those with hypertension are more likely to develop HFS as compared to their non-hypertensive counterparts.

In conclusion, this meta-analysis highlights a positive correlation between hypertension and HFS. Blood pressure should be closely monitored during the follow-up of HFS patients. Preliminary links between VLM compression and HFS should be further evaluation in future studies.

## Methodology

### Systematic Literature Review

Papers were sourced from The Cochrane Library, PubMed, Web of Science as well as Scholar.google.com. We identified 62 papers and eventually shortlisted 7 of these papers based on the following inclusion criteria:

1. Case-Control Study
2. Data publicly available for analysis
3. Sample size of at least 60 HFS cases
4. Published from Year 2000 onwards

We chose to use case-control studies where a clear comparison is made between the prevalence of hypertension in HFS cases and that in the control group. The data could then be used to generate the values to be used in the meta-analysis. While publication bias may arise from only using publicly available data, we ensured that we screened through all the abstracts, even those from journals which were not publicly available, and found that none of these studies were relevant to our topic. 2 papers^11^,^18^ were subsequently further excluded since their measure of hypertension was indirect. Hence, data from 6 papers were eventually pooled together and analysed.

### Meta-analysis

Meta-analysis helps to increase the statistical power of the data, thus increasing the accuracy of the results drawn as compared to each individual study. This is primarily achieved through increasing the sample size, by combining the data from multiple papers studying the same thing and calculating the weighted average of the results. In addition, by compiling data from these papers and comparing the results on a single Forest plot, convergences and divergences between the results from different papers are represented clearly in a visual format and further analysis can be made^19^.

Only case-control studies were included and the Odds Ratio reflected on a Forest plot. *Q*-statistic was used to investigate the degree of heterogeneity between studies. The overall inconsistency index, *I*^*2*^-statistic, was evaluated to describe the proportion of total variation caused by heterogeneity. *I*^*2*^-statistic of less than 30% of the variability in point estimate was considered as mild heterogeneity, more than 50% was notable heterogeneity, whereas in between was considered moderate heterogeneity.

## Additional Information

**How to cite this article**: Leong, J.-L. *et al.* Revisiting the link between hypertension and hemifacial spasm. *Sci. Rep.* **6**, 21082; doi: 10.1038/srep21082 (2016).

## Figures and Tables

**Figure 1:**
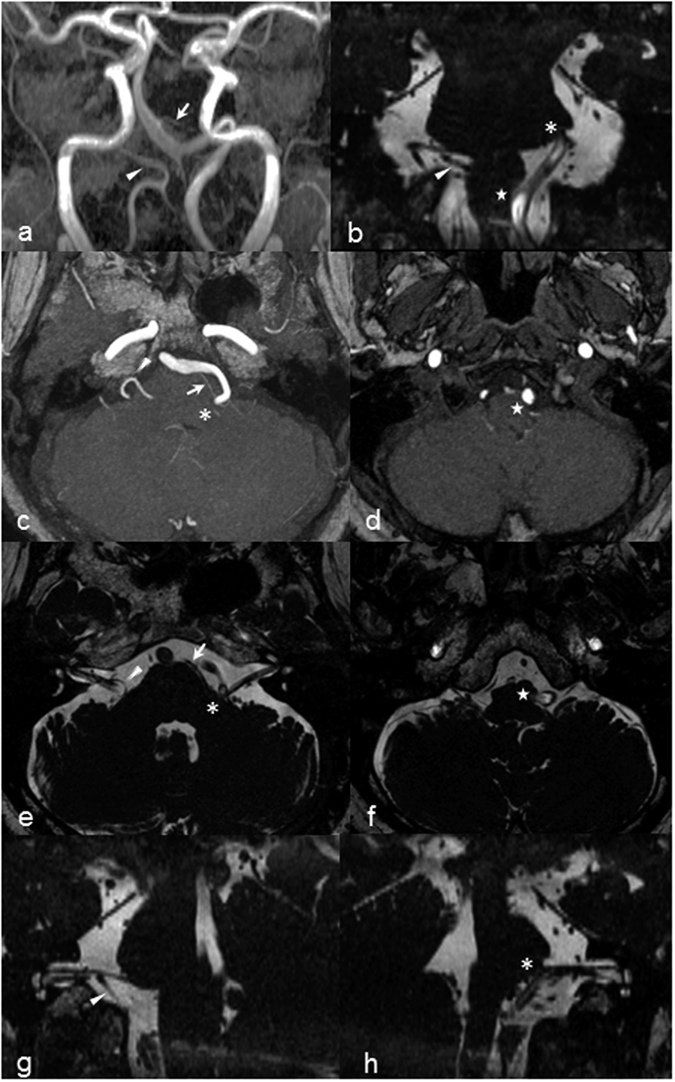
Brain MRI of a hypertensive male with left hemifacial spasm. (**a**) Coronal MRA MIP image showing dolichoectatic of the vertebrobasilar arteries. (**b**) Coronal CISS image showing compression and indentation on the left facial nerve REZ and left VLM by the tortuous left VA. Axial MRA MIP images showing vascular compression at the level of the (**c**) pons by the left VA and AICA, and (**d**) medulla by the left VA. Corresponding axial CISS images detailing compression at the left (**e**) facial nerve REZ and (**f**) VLM. Oblique sagittal reconstructed CISS images along the facial nerve on the (**g**) right showing normal lie of the nerve with PICA nearby and pregnant pontine belly, and (**h**) left showing pontine distortion and elevated facial nerve REZ. (Abbreviations: AICA = anterior inferior cerebellar artery; CISS = constructive interference at steady state; MIP = maximum intensity projection; MRA = magnetic resonance angiography; PICA = posterior inferior cerebellar artery; REZ = root exit zone; vertebral artery = VA; VLM = ventrolateral medulla. Annotations: arrow = left AICA ; arrowhead = right PICA, asterisk = REZ of left facial nerve; star = left VLM compression).

**Table 1:** Demographics of study subjects.

Study ID	Number of participants		Gender (M/F, %)		Age Range, years		Prevalence of HTN, %	
	Cases	Controls	Cases	Controls	Cases	Controls	Cases	Controls
Defazio *et al.* (2000)	115	115	36.5/63.5	36.5/63.5	61.1 ± 10.6	60.8 ± 10.8	40.0	25.0
Defazio *et al.* (2003)	114	228	35.1/64.9	35.1/64.9	20–83	18–84	54.0	38.0
Nakamura *et al.* (2007)	82	82	31.7/68.3	31.7/68.3	44–78	39–83	39.0	29.3
Tan *et al.* (2003)	117	235	36.8/63.2	37.9/62.1	21–80	27–82	42.7	39.1
Sandell *et al.* (2013)	61	50316	62.3/37.7	45.3/54.7	36–75	>20 (upper limit not stated)	31.1	15.4
Rudzinska *et al.* (2014)	60	60	30/70	38/62	58.3 ± 9.1	60.3 ± 10.9	61.6	41.0
